# Using Species Distribution Model to Estimate the Wintering Population Size of the Endangered Scaly-Sided Merganser in China

**DOI:** 10.1371/journal.pone.0117307

**Published:** 2015-02-03

**Authors:** Qing Zeng, Yamian Zhang, Gongqi Sun, Hairui Duo, Li Wen, Guangchun Lei

**Affiliations:** 1 School of Nature Conservation, Beijing Forestry University, Beijing, China; 2 Office of Environment and Heritage, Sydney, Australia; Trier University, GERMANY

## Abstract

Scaly-sided Merganser is a globally endangered species restricted to eastern Asia. Estimating its population is difficult and considerable gap exists between populations at its breeding grounds and wintering sites. In this study, we built a species distribution model (SDM) using Maxent with presence-only data to predict the potential wintering habitat for Scaly-sided Merganser in China. Area under the receiver operating characteristic curve (AUC) method suggests high predictive power of the model (training and testing AUC were 0.97 and 0.96 respectively). The most significant environmental variables included annual mean temperature, mean temperature of coldest quarter, minimum temperature of coldest month and precipitation of driest quarter. Suitable conditions for Scaly-sided Merganser are predicted in the middle and lower reaches of the Yangtze River, especially in Jiangxi, Hunan and Hubei Provinces. The predicted suitable habitat embraces 6,984 km of river. Based on survey results from three consecutive winters (2010–2012) and previous studies, we estimated that the entire wintering population of Scaly-sided Merganser in China to be 3,561 ± 478 individuals, which is consistent with estimate in its breeding ground.

## Introduction

Knowledge of population and distribution is critical for assessing the conservation status of a species, which is often a priority for successful management and conservation [1]. The IUCN Red List is widely used to check the status of species populations and to inform policy and conservation [2]. While the IUCN Red List relies on quantitative criteria for listing species, the conservation status of some species is largely informed by expert opinions [3] due to the coarse estimates of distribution range and population size [4]. World Population Estimation (WPE) from Wetlands International uses similar data sources and its quality varies from no estimate, best guess, expert opinion to census based [5]. Bird populations of various species have been identified based on their breeding ranges [5]. For migratory waterbirds, especially for the rare and endangered ones [6, 7], accuracy of population estimates vary greatly between their breeding and wintering areas [8].

The Scaly-sided Merganser *Mergus squamatus*, also known as the Chinese Merganser, is an endemic species restricted to eastern Asia, and is listed as endangered worldwide [9]. Scaly-sided Merganser breeds in south-east Russia and northeast China. Small numbers of wintering birds occur on rivers and fresh water bodies in southern and central China [10–12], and a few individuals overwinter in Japan, South Korea and Taiwan, with occasional records from Myanmar, Thailand and northern Vietnam [10]. No subpopulations are defined for this species. Population estimates are inconsistent ranging from 2,500 up to 10,000 individuals [5, 9, 13, 14]. In recognition of such uncertainties, IUCN recommends further study and verification [9], and researchers are currently trying to locate “the missing birds” in its wintering sites [15].

In this study, we aimed: 1) to address the uncertainty and inconsistency in the estimates of the suitable wintering habitat and population size of the Scaly-sided Mergansers through methodology innovation, and 2) to provide reliable and verifiable estimation of the population size of Scaly-sided Merganser. We first collated the Scaly-sided Merganser wintering occurrence records from our own surveys, bird watch groups and literatures, and screened the recorded for reliability and accuracy through expert consultation and observer interview. We then modelled the wintering distribution of the species using Maxent, a maximum-entropy approach for species habitat modeling [16]. With the predicted suitable wintering areas, we calculated the wintering population of the Scaly-sided Merganser in China using bird density estimated from systematic field survey data.

## Materials and Methods

This study was authorized by Department of Forestry of Jiangxi Province and Department of Forestry of Hunan Province.

### Scaly-sided merganser occurrence data

**Figure 1:**
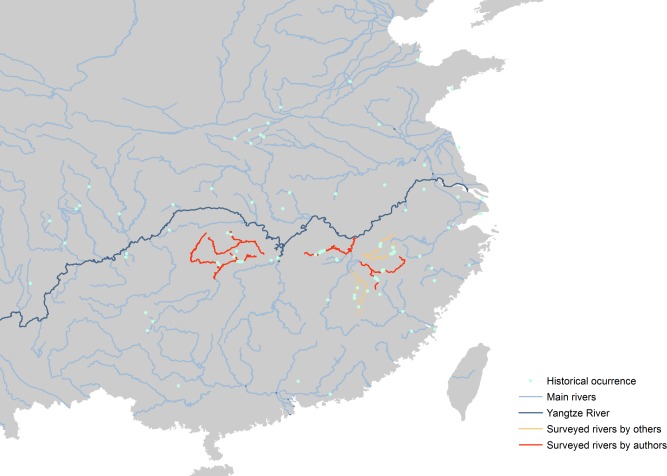
Surveyed rivers in distribution areas of Scaly-sided Merganser. From left to right are Li River, Yuan River, Xiu River, Fu River, Xin River, Rao River.

We compiled 100 geo-referenced sighting records from our surveys in three winters (2010–2012), literature review [17–20], and from other publicly accessible databases including specialized organization reports, websites of local government and newspaper, and personal communication with experts (Fig. 1 and S1 Dataset). The dataset includes occurrences in the mainland China only. Records without geographic coordinates were examined in GoogleEarth after consulting with the reporters. In addition, sightings at migratory stopover sites were excluded. The cleaned records were arranged in a manner appropriate for input into the Maxent package as an excel file. In species distribution modeling, a common issue with rare species is the small numbers of occurrence records [21]. We considered the 100 records of Scaly-sided Mergansers were sufficient for machine-learning methods, with which average accuracy was near maximal at 50 data points [22].

### Mapping the environmental variables

We used 19 bioclimatic and 4 environmental variables (i.e. elevation, land cover, river density and human disturbance) as predictors. The bioclimatic variables were generated through interpolation of monthly mean temperature and rainfall data from weather stations for the period of 1950–2000 [23], and available for public use at http://www.worldclim.org. The elevation variable was derived from STRM30 dataset (http://srtm.csi.cgiar.org/), land cover was obtained from Global Land Cover database [24], and river density was retrieved from HydroSHEDS dataset (http://hydrosheds.cr.usgs.gov). Human influence was weighed by HII (Human Influence Indicator, Last of the Wild Data Version 2, 2005), produced through incorporating four data types as proxies: human settlement, land transformation, accessibility and electrical power infrastructure, with values ranging from 0 to 64, corresponding to no or maximum human influence. All variables were re-sampled to a spatial resolution of 30 arc seconds (often referred to as ‘1 km’ resolution).

### Species distribution model (SDM)

To predict the potential distribution range of the Scaly-sided Merganser, we used maximum entropy implemented in the Maxent package (version 3.3.3k) [25, 26]. Maxent is among the most robust and accurate SDM techniques [25]. In recent years, it has gained popularity in conservation studies partly because the technique is less sensitive to the number of recorded sites [27], and relies on presence-only data. As a machine learning modeling technique, it compares the environmental variables underlying the input points against a random sample of background points that represent the availability and range of environmental conditions, and produces a raster map ranking each cell in the study area with an index representing relative habitat suitability. Maxent accounts for interactions among predictor variables [26] and deals with model complexity by employing the L1-regularization procedure to overcome overfitting [28], thereby can better handle correlated predictor variables comparing with other methods such as generalized additive modelling [26, 27].

In developing the SDM, we set the program to randomly take 75% occurrence records for model training and the remaining 25% for model testing. We replicated the modeling process 30 times to test the model performance and to measure the amount of variability in the model. We used the average model for the subsequent suitable habitat calculation. We used the mean area under the receiver operating characteristic curve (AUC) to evaluate the model performance. A random prediction will result in an AUC value of 0.5 whereas a perfect prediction assumes the maximum possible AUC of 1.0 [29], and AUC values > 0.75 are considered as suitable for conservation planning [30, 31]. AUC has been increasingly used in the evaluation of models of species distributions [32] and was regarded as one of the best assessments [33] with the primary advantage of providing a single measure of model performance, independent of any particular choice of threshold [16, 25]. In addition, we used AUC-based test to assess the contributions of environmental variables considering that it corrects (to some degree) the bias and is better than the standard percent contribution [34]. We rerun the model without the variables with permutation importance less than 1%.

A specific threshold is needed to transform the model results of logistic occurrence probabilities or suitability index to presences/ absences [35]. However, the fixed threshold approach (e.g. 0.5 [36], 0.3 [37], 0.05 [38]) is arbitrary and lacks ecological basis [39]. Average predicted probability was used as it is considered a more robust approach [40]. Areas with an occurrence probability above the threshold are regarded as modelled distribution areas for Scaly-sided Merganser.

### Suitable habitat size

The predicted suitable habitat from Maxent was reclassified in ArcGIS 9.3 with Spatial Analyst tools. We then converted the reclassified raster to a polygon shape file. To calculate the size of suitable habitats, we overlaid the suitable habitat polygons with river polylines and summed up all intersected river length. Small rivers of order five and below were excluded in calculation because Scaly-sided Mergansers are generally absent in these environments according to historical record of occurrence (Fig. 1). In addition, during the three-year survey, we did not find any Scaly-sided Merganser in small rivers (i.e. order five and below) within the known distribution regions. The high-level human disturbance might prevent the presence of Scaly-sided Mergansers at smaller rives in China.

### Field survey of population density & population size estimation

Based on historical occurrence data, four representative rivers in distribution area were chosen for field survey during 2010–2012 (Fig. 1). The Li River and Yuan River represent the marginal distribution area, whereas the Xiu River and Xin River represent the central distribution area of Scaly-sided Merganser according to historical sighting records. In each river, we counted all Scaly-sided Merganser at the surveyed continuous sections by boat or walk using line transect sampling. Combined with the data from previous studies [19, 20] in other rivers (Fig. 1) within the distribution area, we calculated the population density of Scaly-sided Merganser.

While the width of these rivers in distribution area could vary from 80–700 m [41], length was considered to the most suitable to calculate population density for rivers. We estimated the potential Scaly-sided Merganser population by projecting these observed population densities in the main known habitat, the middle reaches of Yangtze River with reference to the entire modelled distribution area [42, 43]:

$${\text{N}}_{\text{c}}=\;{\text{D}}_{\text{r}}*\;\text{L}$$

where:

N_c_ is estimated population of Scaly-sided Merganser,

D_r_ is density of Scaly-side Merganser in rivers,

L is length of river sections in modelled presence areas.

## Results

### The Maxent species distribution model

**Figure 2:**
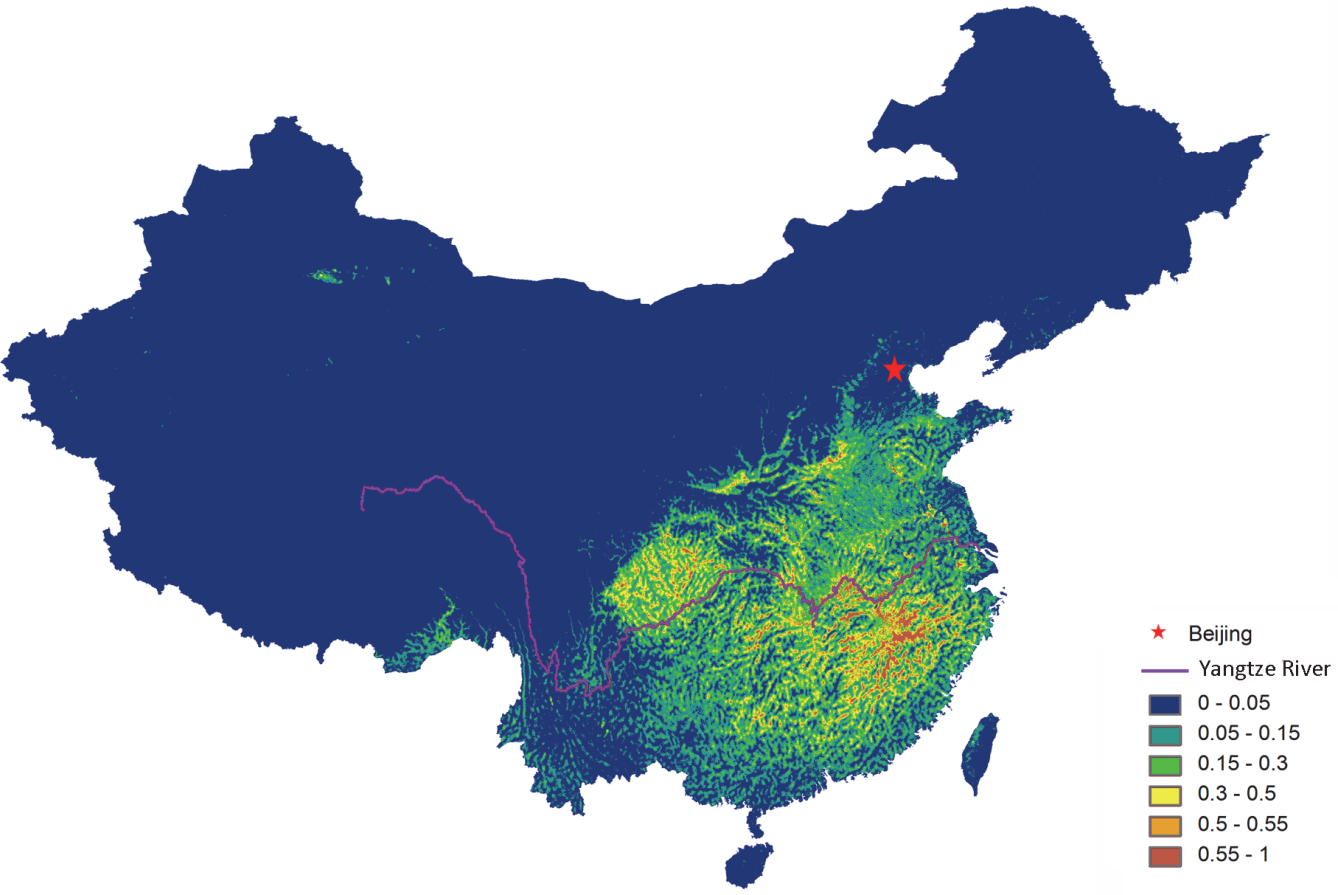
Potential wintering distribution areas for Scaly-sided Mergansers in China. Red indicates predicted distribution area with high occurrence probability.

The map of potential habitat shows that highly suitable habitats for Scaly-side Merganser are located in the middle and lower reaches of the Yangtze River (Fig. 2), especially in Jiangxi, Hunan and Hubei Provinces. Sichuan, Chongqing, and Henan Province also have areas of high importance for Scaly-sided Merganser.

The average training AUC for the replicate runs is 0.972 (0.961 for testing runs), and the standard deviation of the 30 runs is very small (0.004 and 0.009 for training and testing) which suggest excellent predictive power of the fitted model [30] compared with the value (0.5) expected from a random prediction.

**Figure 3:**
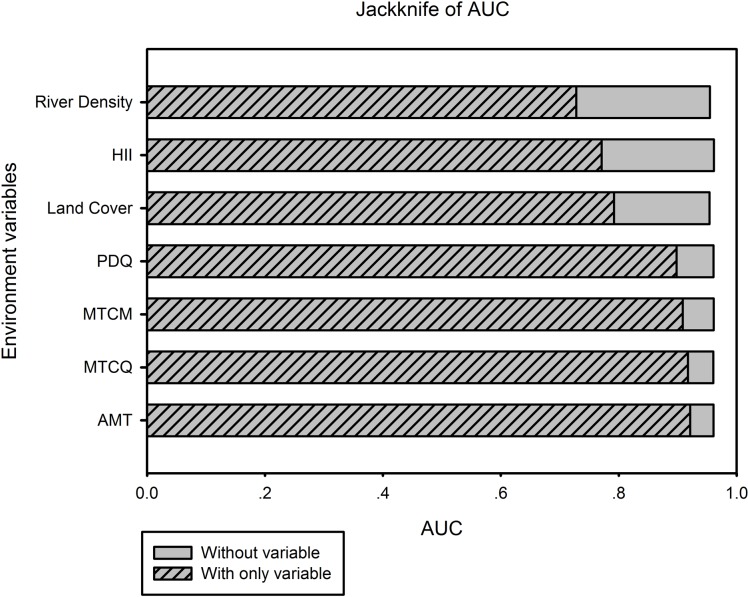
Jackknife test of AUC for envrionmental variables. HII = Human Influence Indicator, PDQ = precipitation of driest quarter, MTCM = minimum temperature of coldest month, MTCQ = mean temperature of coldest quarter, AMT = annual mean temperature.

Annual mean temperature, mean temperature of coldest quarter, minimum temperature of coldest month and precipitation of driest quarter are the most important variables in the Scaly-sided Mergansers occurrence (Fig. 3). Stream density, human influence and land cover are factors of lesser importance (Fig. 3).

**Figure 4:**
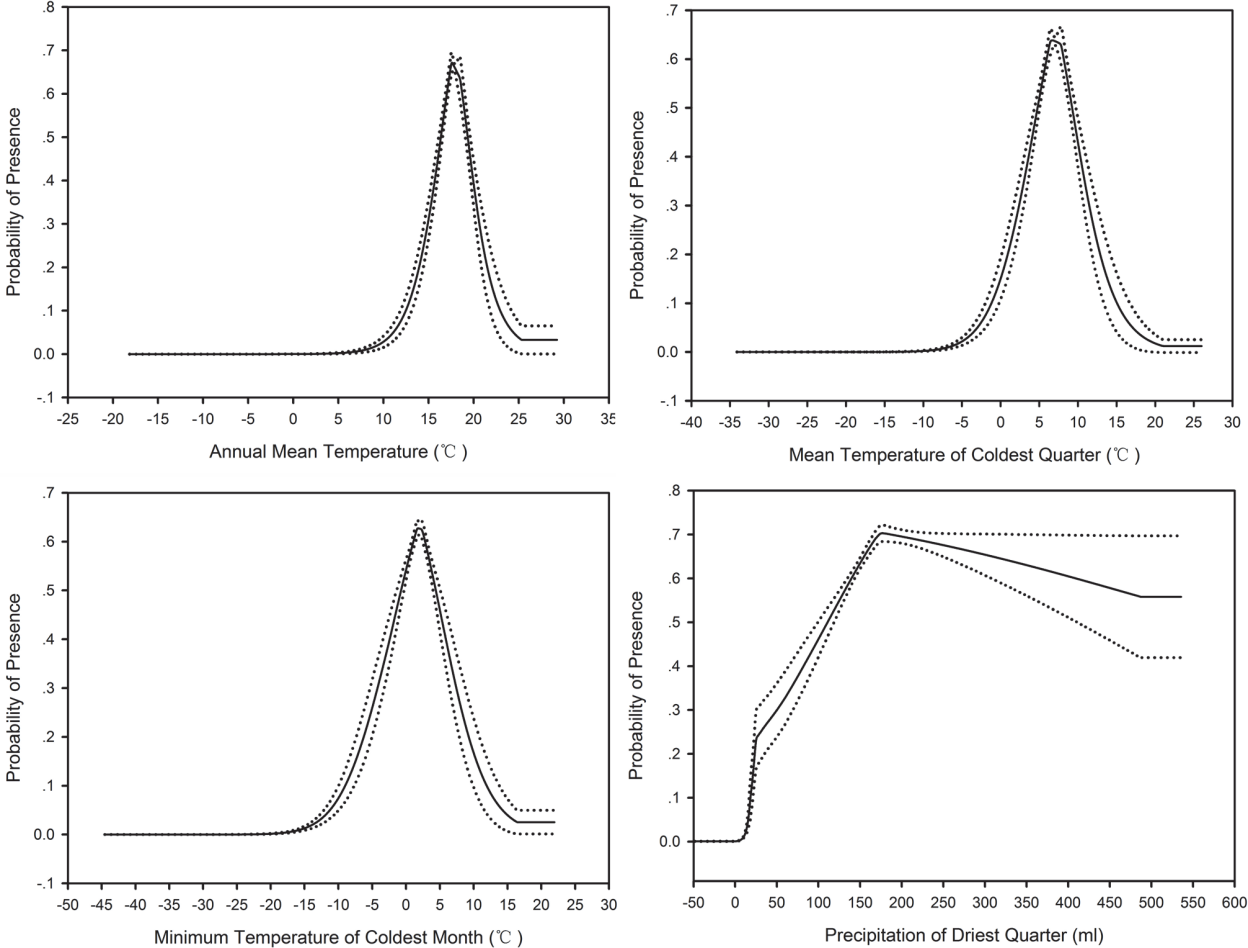
Response curves of environmental variables with highest contribution to occurrence of Scaly-sided Merganser. Solid line is the mean response curve and dotted lines are ±1 SD.

The partial response curves of the four highly influential variables were presented in Fig. 4. The strong non-linear relationships between the probability of Scaly-side Merganser occurrence and predictor variables are evident. The most suitable habitat for Scaly-side Mergansers are areas with annual mean temperature around 17°C, mean temperature in winter around 6°C minimum temperature in coldest month around 2°C, and winter precipitation around 170 mm.

### Population Estimates

Combined with our ground surveys during 2010–2012 and previous studies in the main rivers within the distribution areas, density of Scaly-sided Mergansers in distributed river varied from 0.4–0.61 individual per km (Table 1). The average predicted probability in Maxent is 0.55, and the total length of river segments with above 0.55 occurrence probability was 6,984 km. Therefore, the population of wintering Scaly-side Mergansers in China was estimated to be 3,561 ± 478 individuals.

**Table 1:** Surveys in main rivers in distribution areas and population density of Scaly-sided Mergansers.

**River**	**Section**	**Length (km)**	**Population**	**Density (per km)**
Li River	Xieshui	47	27	0.57
Yuan River	Youshui	30	12	0.40
	Taoyuan	99	42	0.42
Xiu River	Upper reach	71	43	0.61
	Middle reach	63	32 [20]	0.51
Fu River	Yihuang	65	31 [20]	0.48
Xin River	Luxihe	43	23	0.53
Rao River	Leanhe	57	31 [19]	0.54

## Discussion

### Potential habitat

Bird watchers and researchers might be more likely to visit and revisit areas that are considered to be rich in species [44], therefore we acknowledge that sample bias [45, 46] resulting from survey accessibility might exist, especially when it comes to Scaly-sided Mergansers, which are relatively small in size, occur in small flocks, and are few in number, and thus have a lesser chance of being noticed. Nevertheless, the predictions for winter distributions suggest a good agreement with historical occurrence records. Besides the known presence hotspots in Jiangxi Province, our SDM suggested that some rivers in Hunan, Hubei, Sichuan, Chongqing, Henan Province were highly suitable habitats for the endangered duck (Fig. 2). And more rivers should be systematical surveyed in the future conservation planning including: Du River in Hubei Province, Xiang River, Chen River, Wu River in Hunan Province, Zitong River, Qu River, Zhou River in Sichuan Province, Jin River in Guizhou Province, Luoqing River, Gongcheng River in Guangxi Province, Qiupu River in Anhui Province, and Jin River, Futun River, Nanpu River in Fujian Province.

### Environmental variables

It has been widely recognized that climate (temperature, precipitation, etc.) had strong influences on bird distribution and richness [47]. In consistence with this, our results showed that the annual mean temperature, in particular, the minimum temperature of coldest quarter exerts strong influence in the probabilty of the occurrence of the wintering Scaly-sided Merganser. Similarly, the population growth of White-throated Dipper *Cinclus cinclus* in non-breeding season was influenced by mean winter temperature and precipitation in both regional and local scale during a 31-year study [48]. Winter weather conditions play a predominant role in the distribution of Scaly-sided Mergansers because they primarily depend on open water in river for foraging, and lower temperature could bring large ice coverage and therefore decrease the foraging area. In addition, windy and raining condition could affect the foraging efficiency, and the spatial distribution of birds [49].

In a distribution modeling study on Black-faced Spoonbill *Platalea minor*, Hu *et al.* [35] excluded six variables directly connected to summer condition. In our study, however, the Jackknife tests indicated that most of the investigated environmental variables contributed to the model with various degree of importance. We did not rerun model with refined winter-only variables, considering effect of summer conditions on hydrology and vegetation. Furthermore, hydrology and geomorphology interact to produce a heterogeneous thermal template for natural selection influencing fish spawn timing across river basins [50], and fish are major food source for Scaly-sided Merganser in winter.

Land cover, river density and human influence (HII) had less contribution than the bioclimatic variables. The results are in agreement with studies in the Russian breeding grounds, where river size, mountain slope, estimated forest cover and human population all failed to explain the observed distribution [51]. The effect of human influence can be immense, yet our understanding is rudimentary. There is considerable uncertainty about how and why animals are disturbed by humans [52], and the most likely reason is that wild animals perceive humans as potential predators and respond accordingly [53]. However, in some urban wetlands, intrusion of humans or domestic dogs had no measurable negative effect on endangered species occurrence [54]. Furthermore, there are debates on whether HII is a “good” indicator of human disturbance to wildlife, although Hu *et al.* [35] found that HII contributed the most in the distribution of Black-faced Spoonbill. Firstly, the dataset was integrated in 2005, while big construction projects (e.g. road, dam) springing up with the rapid economic development, and there might be some gaps between HII dataset and actual condition. In addition, human settlement and accessibility are generalized concepts and perform with low consistence, especially for waterbirds, whereas rivers work as buffer and may keep species away from human interference. In fact, there are a number of records of Scaly-sided Merganser in urban river across cities. Another proxy of the HII, electrical power infrastructure, also served ambiguously to weigh human influence in our study. We found that Scaly-sided Mergansers preferred river sections not far downstream of dams while they were rarely observed in the upstream reaches during field surveys.

### Population estimates

The accuracy of population estimation is closely tied to good monitoring design, and long-term systematic data collection. However, frequently used methods include mark-resight, capture-recapture with robust design (closed capture) models [55] and long-term censuses such as aerial surveys [56] are resource-demanding, and are seldom employed for a single species. In addition, conventional visual estimation of rare species may not always be conclusive, both underestimation [8] and overestimation [57] were noted. We estimated wintering population size of Scaly-sided Merganser by extrapolating from observed densities in main rivers after adjusting for habitat suitability. Field survey covering larger area could be used to fine tune the density estimation. Our population estimate represents a relatively reliable approximation rather than an accurate census of the species. Newly published population estimates for land birds [58] suggest that transferring probability into encounter rates to obtain density was plausible. Our study followed it in principle by overlying the polyline features of rivers over the suitable habitat polygons because the major habitats of Scaly-sided Mergansers are linear river systems.

A comprehensive population estimate requires not only determining breeding population size but also considering the non-breeding populations, which may be smaller after long-distant migration. Recently, much attention has been paid to the role of the population at the non-breeding stage [59]. Our estimate of 3561 ± 478 of the Chinese wintering population is closer to that of the IUCN Red List, but much less than upper bound of WPE 5 estimates (i.e. 10,000). One reason may be that the IUCN Red List counts only breeding pairs while in WPE 5, breeding pairs are multiplied by three to give the total population size [5]. Solovyeva *et al.* [60] estimated the global population to be c.1940 pairs (c. 4660 birds) prior to reproduction. There is reasonable concordance between the two estimates, and would be more consistent if wintering populations in the Peninsula of Korea were included. Nail *et.al* [61] counted 149 birds in the Republic of Korea, and Duckworth and Chol [62] recorded more than 40 birds in central Korea. Therefore the wintering population of Scaly-sided Mergansers in Korean Peninsula would be close to a few hundreds.

Studies suggested that 500 individuals were required to maintain the evolutionary potential of the species [4, 63] and an overall population of 5,000 individuals would be required to prevent the loss of quantitative genetic variation in a species [64]. Thus, our findings indicated that the Scaly-sided Merganser was still endangered and the survival of the species requires conservation efforts on an international scale.

## Supporting Information

S1 Dataset Historical occurrence dataset for wintering Scaly-sided Merganser in China. (XLSX)
